# The Relation of Over-Nutrition, after the Acute Fevers of Childhood, to Bone Disease

**Published:** 1885-07

**Authors:** 


### The Relation of Over-Nutrition, after the Acute Fevers of Childhood, to Bone Disease.

Under this title there is reported in the *Cincinnati Obstetric Gazette*, for September, a summary of the remarks made on this subject by Dr. A. Jacobi at a meeting of the New York Obstetrical Society.

